# Supplementary figures and images for: Determinants of early mental health help-seeking among women in Bangladesh: A nationally representative bootstrapped regression analysis
Source: PLOS Ment Health. 2025 Sep 12;2(9):e0000420. doi: 10.1371/journal.pmen.0000420 (PMC12798417; doi:10.1371/journal.pmen.0000420)

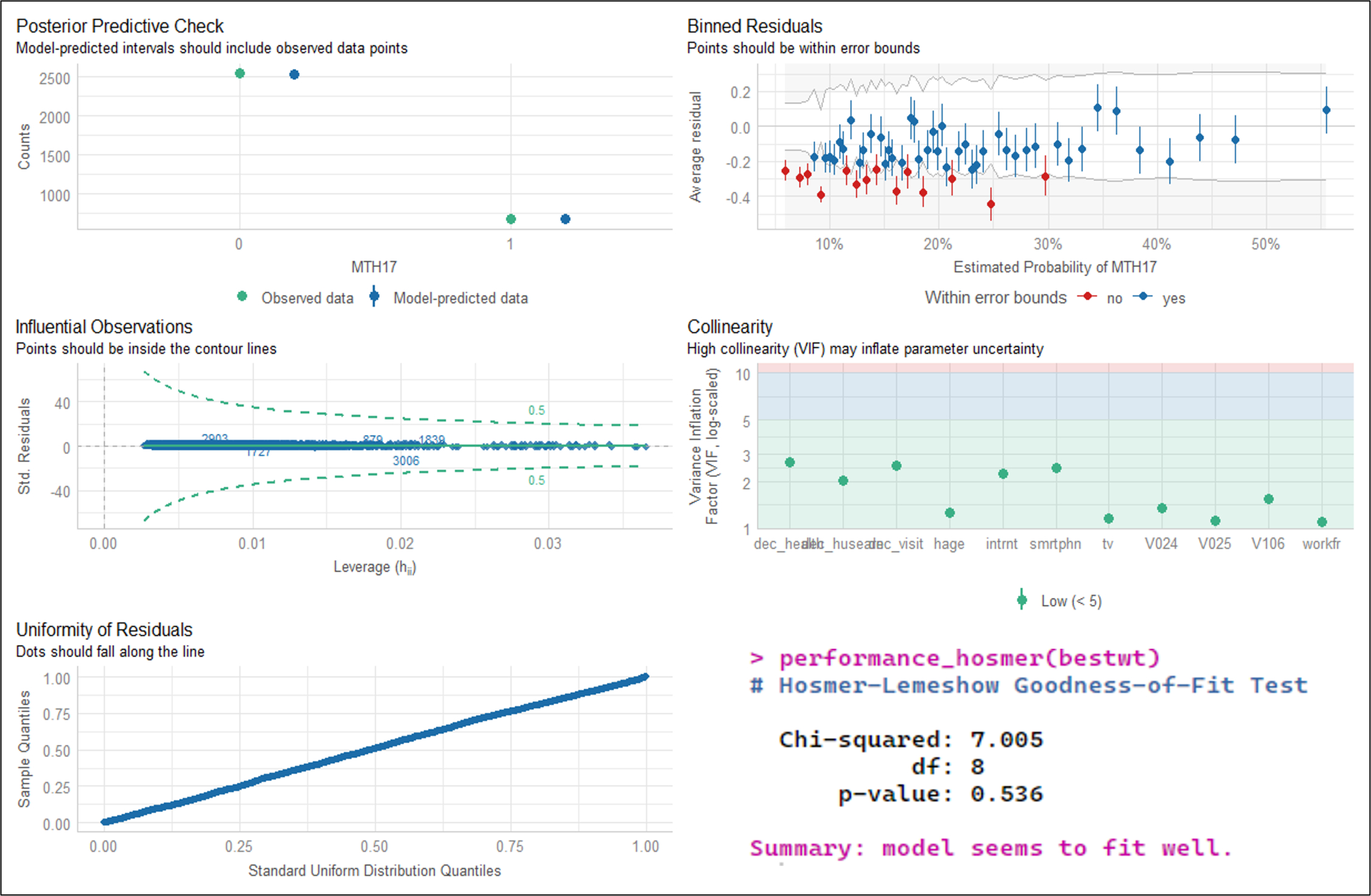

Supplement: S1 Fig — (TIF) [file pmen.0000420.s001.tif]

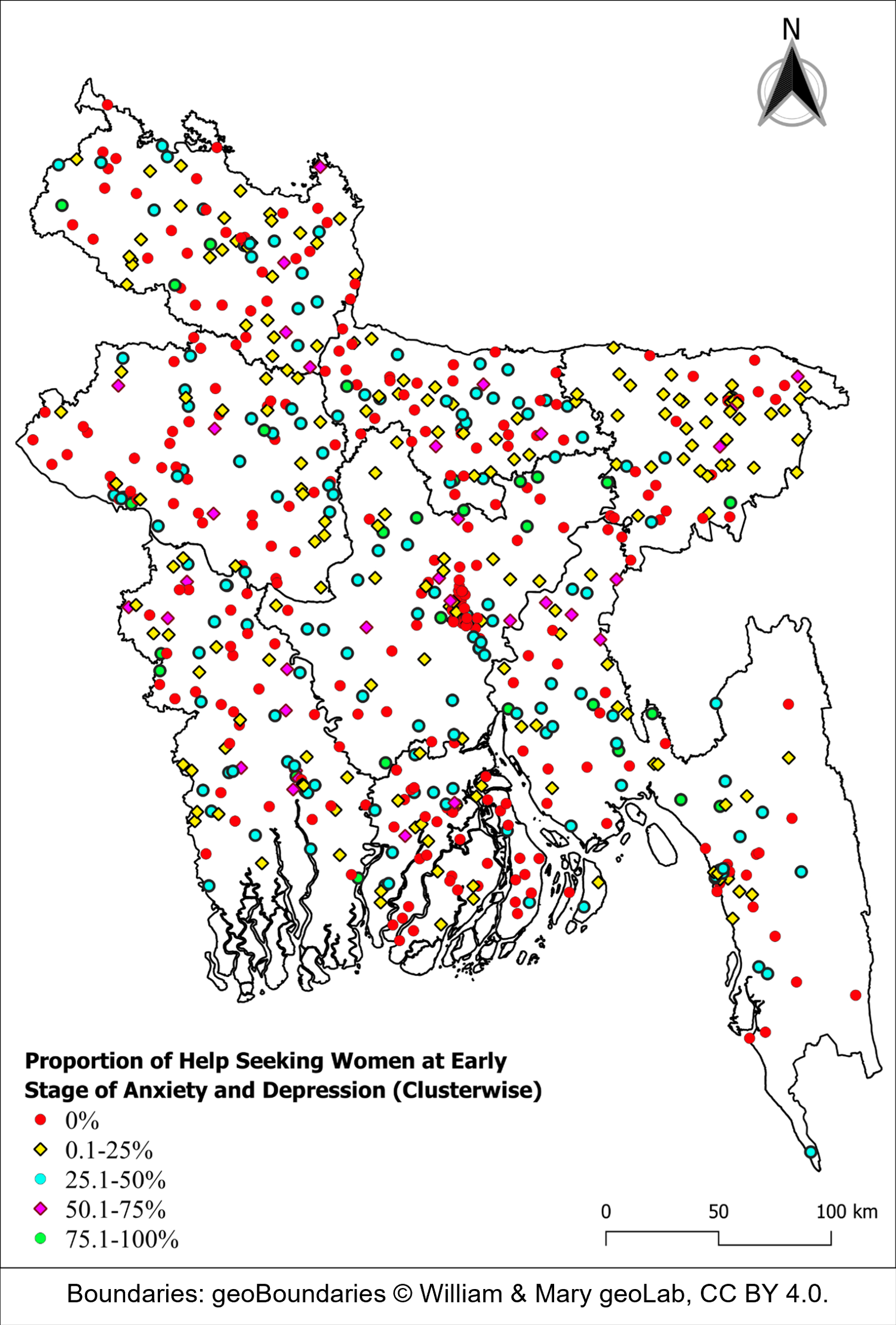

Supplement: S2 Fig — (TIF) [file pmen.0000420.s003.tif]
