# Supplementary material for: Determinants of early mental health help-seeking among women in Bangladesh: A nationally representative bootstrapped regression analysis
Source: PLOS Ment Health. 2025 Sep 12;2(9):e0000420. doi: 10.1371/journal.pmen.0000420 (PMC12798417; doi:10.1371/journal.pmen.0000420)
Supplement: S1 File — (DOCX) [file pmen.0000420.s002.docx]

Exploration of the Possible Random effect model:


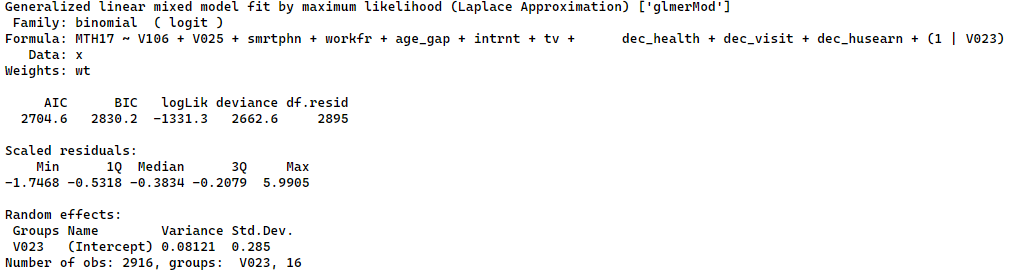


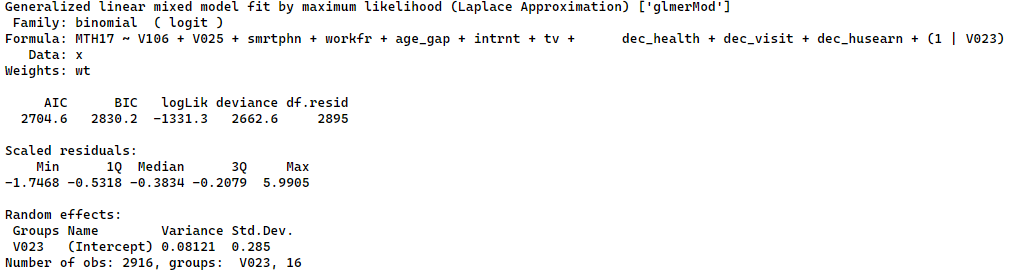


keeping Strata (V023) as random intercept (assuming baseline odds of help seeking varies across rural and urban residences of different divisions) showed small improvement (ICC = 0.024) in variance over the fixed effect model’s variance.

| **S1 Table: Intercept (Baseline Odds of Help Seeking) of Each Strata** | | | | | | |
| --- | --- | --- | --- | --- | --- | --- |
|  | **Urban** | | | **Rural** | | |
| **Strata** | ***Estimate*** | ***CI_lower*** | ***CI_upper*** | ***Estimate*** | ***CI_lower*** | ***CI_upper*** |
| Barisal | *1.006716792* | *0.615688301* | *1.646090559* | *0.700482542* | *0.488358726* | *1.004744599* |
| Chittagong | *1.225246194* | *0.887363821* | *1.691784362* | *1.215407143* | *0.979555448* | *1.508045844* |
| Dhaka | *0.795698717* | *0.444590855* | *1.198165228* | *1.113697661* | *0.872521068* | *1.421538716* |
| Khulna | ***1.609609859*** | *1.11790786* | *2.317582683* | ***1.31543886*** | *1.026948198* | *1.684972424* |
| Mymensingh | *0.932843157* | *0.572541868* | *1.519882482* | *0.95881635* | *0.693910258* | *1.324852577* |
| Rajshahi | 1.189170248 | 0.814539773 | 1.736104147 | 0.980672487 | 0.766124413 | 1.255303329 |
| Rangpur | 0.886236992 | 0.554460001 | 1.416542229 | 0.950882194 | 0.755787954 | 1.196336806 |
| Sylhet | 0.951853517 | 0.586062625 | 1.545952733 | 0.960596104 | 0.671149561 | 1.374872204 |

Only Khulna had a significantly higher baseline odds in both rural and urban settlement.


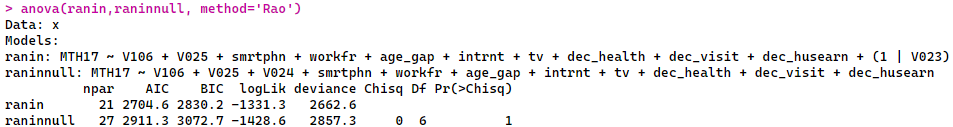


Random Intercept model showed no significant improvement compared to the fixed effect model


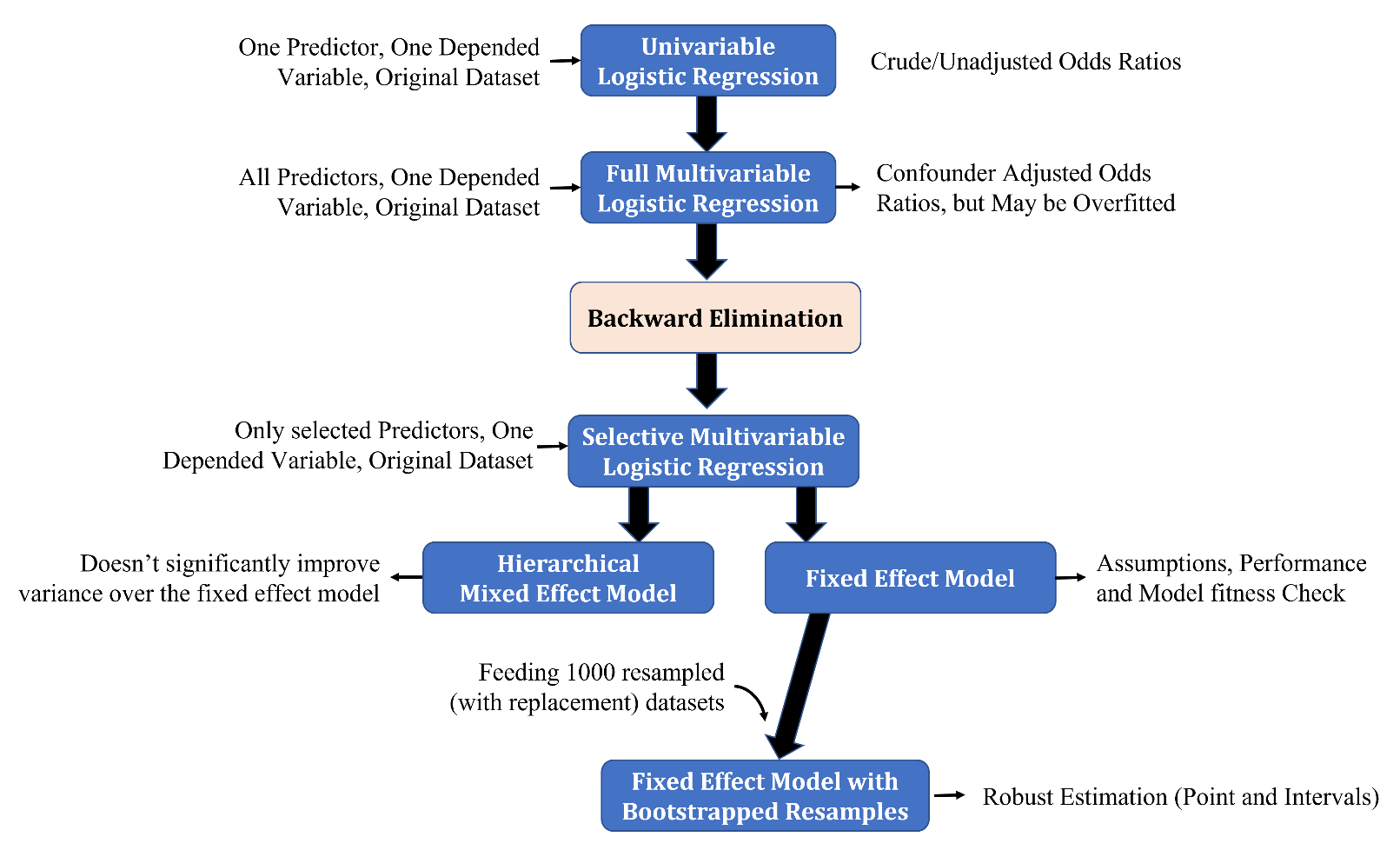


S1 Fig. Analysis Pathway for this Study


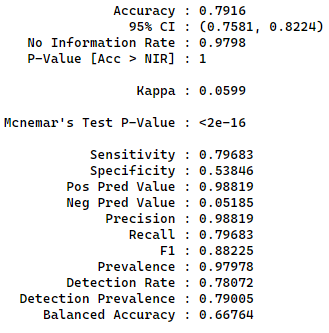

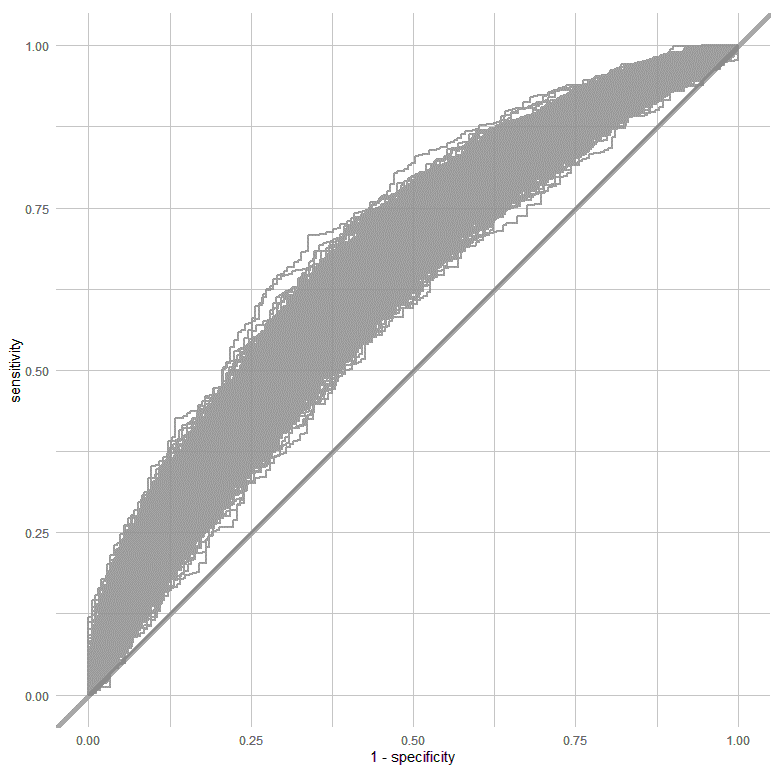


AIC = 2911

McFadden’s R2 = 0.23

Log_Loss = 0.48

S2 Fig. ROC of Bootstrapped Logistic Regression Models

| **S2. Table: Model Metrices** | | | | | |
| --- | --- | --- | --- | --- | --- |
| .metric | .estimator | .estimate | .standard_error | n | .config |
| accuracy | binary | 0.79 | 0.0004 | 1000 | Preprocessor1_Model1 |
| roc_auc | binary | 0.67 | 0.0006 | 1000 | Preprocessor1_Model1 |
| brier_score | binary | 0.14 | 0.0002 | 1000 | Preprocessor1_Model1 |

S2 Fig. Assumption Assessment of the Initial Model before Bootstrapping

Dataset available at: [The DHS Program - Available Datasets](https://dhsprogram.com/data/available-datasets.cfm)

Analysis Script can be accessed by reasonable request to corresponding Author (R Script)

Fig 06. Interpolated Proportion of Women Sought for Help

Boundaries: geoBoundaries © William & Mary geoLab, CC BY 4.0.


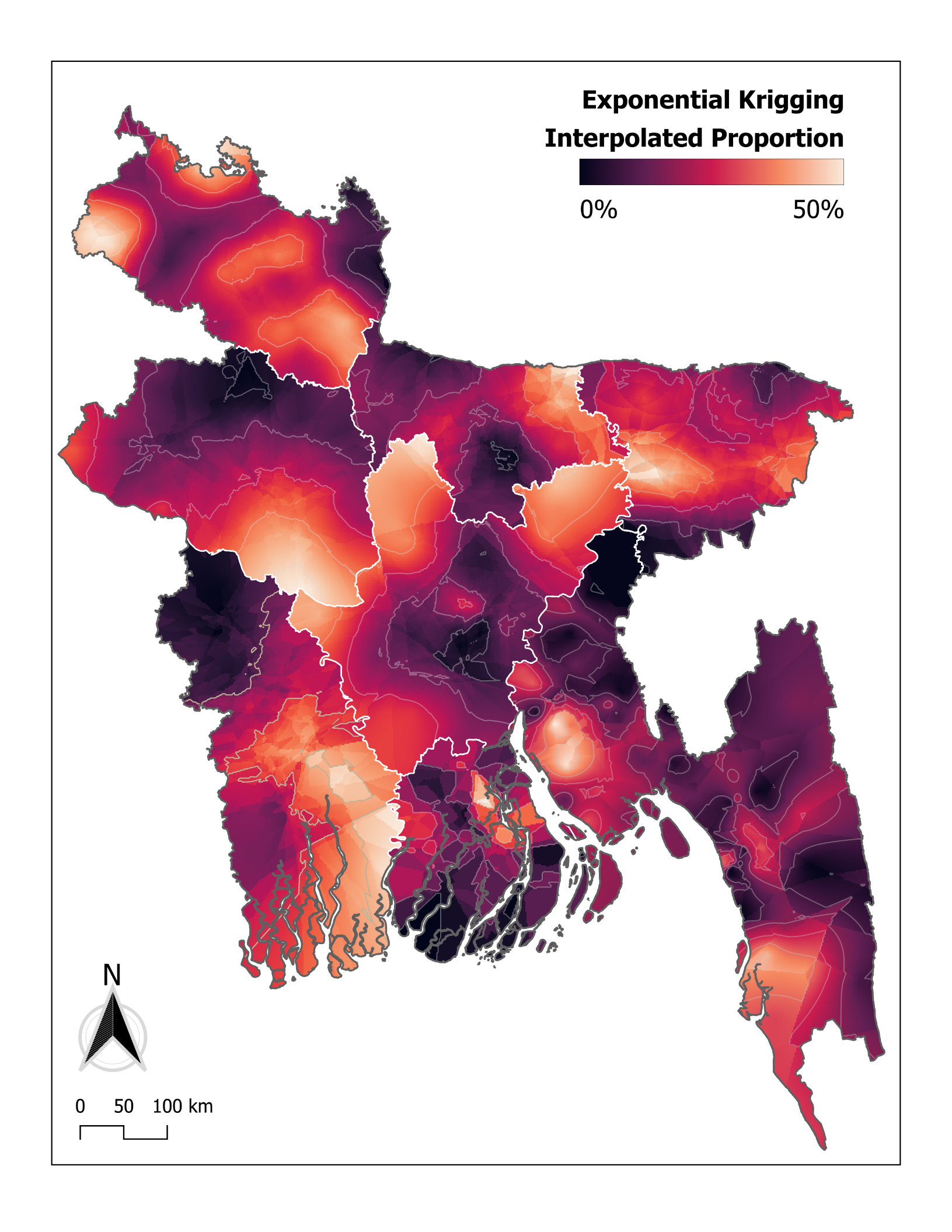


in Early Stage of Depression and Anxiety by Exponential Krigging
