# Supplementary material for: Determinants of early mental health help-seeking among women in Bangladesh: A nationally representative bootstrapped regression analysis
Source: PLOS Ment Health. 2025 Sep 12;2(9):e0000420. doi: 10.1371/journal.pmen.0000420 (PMC12798417; doi:10.1371/journal.pmen.0000420)
Supplement: Attachment — Submitted filename: Response to Reviewers.docx [file pmen.0000420.s005.docx]

Dear Editors and Reviewers,

We are grateful to you for investing your valuable time on reviewing our manuscripts. We have carefully read your comments and addressed accordingly where most of the co-authors agreed that it is necessary. We have also decided not to change our manuscript as exactly as some of the comments and the reasons are explained below.

We want to sincerely thank you again for your comments that will strengthen our manuscript.

Dr. Tanvir Ahmed

Corresponding Author

# **Response to Editor:**

**#1.** Please provide separate figure files in .tif or .eps format.

Our Response: Image files are now Submitted according to the desired format.

**#2.** We have amended your Competing Interest statement to comply with journal style. We kindly ask that you double check the statement and let us know if anything is incorrect.

Our Response: Everything is all okay from us.

**#3.** If you did not receive any funding for this study, please simply state: “The authors received no specific funding for this work.”

Our Response: We have simplified that section in revised version.

**#4.** Thank you for uploading your study's underlying data set. Unfortunately, the repository you have noted in your Data Availability statement does not qualify as an acceptable data repository according to PLOS's standards.

Our Response: Datasets are now uploaded in figshare with public DOI provided.

**#5.** Some material included in your submission may be copyrighted. According to PLOS’s copyright policy, authors who use figures or other material (e.g., graphics, clipart, maps) from another author or copyright holder must demonstrate or obtain permission to publish this material under the Creative Commons Attribution 4.0 International (CC BY 4.0) License used by PLOS journals. Please closely review the details of PLOS’s copyright requirements here: PLOS Licenses and Copyright. If you need to request permissions from a copyright holder, you may use PLOS's Copyright Content Permission form.

Our Response: We have used publicly available shapefiles for mapping. We will change the figure 03 accordingly and add legends and citations where necessary. We clarify that we didn’t use any copyrighted images.

**#6.** If the reviewer comments include a recommendation to cite specific previously published works, please review and evaluate these publications to determine whether they are relevant and should be cited. There is no requirement to cite these works unless the editor has indicated otherwise.

Our Response: Duly addressed in the revised manuscript.

# **Response to Reviewers:**

## **Comments from Reviewer 1:**

**# 1.** Title is accurate but long. Suggest trimming for clarity “Determinants of Early Mental Health Help-Seeking Among Women in Bangladesh: A Nationally Representative Analysis”

Our Response: We have shortened the title according to your suggestion, but added the ‘Bootstrapped Regression’ part as we think it is one of the core strengths of our manuscript to focus on.

**#2.** Abstract: Results can be grouped more concisely, too many aORs may overwhelm readers.

Our Response: We have rewritten some of the lines to reduce aORs and to improve the flow of that section

**#3a**. Some sentences are long and could be broken into simpler, clearer statements (e.g., lines 54-57, 281-283 and 396-399).

Our Response: We have revised our whole manuscript and tried to avoid complex and long sentences overall. We used conjunctions and linking words, instead of punctuations where it deemed needed.

**#3b.** Consider avoiding repetition (e.g., lines 48–57 and 63–66 discuss the burden and importance of early intervention similarly).

Our Response: We did not make any change according to this comment. Our intention is to explain why early intervention is ever so more important in Bangladesh. 63-66 part describes that situation.

**#4a.** Clarify “bootstrapped fixed-effects logistic regression” for general readers. Are the strata treated as fixed intercepts?

Our Response: Yes, DHS uses Divisions and Residence for stratification for sampling, and we kept those as fixed effect in our model. We’ve clarified this now in the analysis part.

**#4b.** Variable selection process ("backward stepwise") is clear but could benefit from a brief note on why it was preferred over LASSO or AIC-based selection.

Our Response: Backward stepwise process can consider one of many criteria to eliminate any variables from the model, we clarify that applied AIC-based backward elimination. Reasons why we didn’t go for LASSO regression -

1. We wanted to retain the actual variables without shrinking the coefficients. We think this will be easier for stakeholders to understand.

2. All the variables of our model are categorical and there’s absolute absence of multicollinearity, so Stepwise is just fine without the shrinkage benefit of LASSO or other regularization method.

3. We were not interested to make a predictor model, rather we focused on explanatory model to get estimates, we thought LASSO was just overkill for this, we still think so.

We have added a brief discussion on the justification for using this method.

**#4c.** Although the manuscript refers to several figures, they are not embedded in the main text. While the figures have been provided as supplementary files, their resolution is too low to interpret the content effectively.

Our Response: For this, We’d like to refer to the “Submission Guidelines” of PLOS Mental Health, Section: Parts of a Submission, Sub section: Figures and Tables ([Submission Guidelines | PLOS Mental Health](https://journals.plos.org/mentalhealth/s/submission-guidelines#loc-figures-and-tables)) that guided us to upload figures as separate files.

And regarding the resolution of the figures, we have revised all the figures as per journal guideline. Some of the figures (S2 Figure and S3 Figure) are uploaded as supporting documents. We did little effort to make them attractive, resolution and file format, perhaps, are matched with the journal guideline now

**#5a.** Clarify the contrast between “Smartphone users” vs. “Internet users”. This is nuanced but may confuse some readers.

Our Response: Not all women who owned a smartphone were internet users. Now clarified in the revision.

**#5b.** Consider summarizing key findings in a sentence or two before launching into detailed odds ratios.

Our Response: Revised accordingly.

**#5c.** Group related predictors (e.g. autonomy-related ones) to reduce the density of Table 03.

Our Response: Autonomy related variables are part of large survey-based women empowerment scale (SWPER), as BDHS didn’t contain all the necessary variables we didn’t include Empowerment variable that is recoded from other variables. Also we didn’t find any particular scale/index reference to group all the autonomy-related variable together, so we kept those as it is. And some of the autonomy related variables (eg. Decision maker of large household purchase) is not related to healthcare decision making, that is properly addressed by our final model, that’s why we still choose to keep them as they are.

**#6.** Some sections lean toward advocacy without adequate empirical support. For instance, the suggestion to train housewives as peer counselors (lines 412–414) is conceptually valuable but would benefit from supporting evidence or citations. Similar concerns apply to the strong recommendations presented in lines 399–403 and 414–416, which would be more compelling if grounded in existing research or programmatic examples.

Our Response: Direct evidence of the impact interventions is yet to be established (like impact of TV programs or peer support on mental health), We thought this was a major gap in current mental health situation. Because these types of interventions have already brought positive results in different domains of health prevention, promotion and practice.

1. Evidence from diverse low-resource (Bangladesh, Tanzania, Uganda) settings has demonstrated that peer counseling by laywomen can effectively improve health behaviors and psychosocial outcomes, particularly when rooted in shared lived experiences and community trust.^1–4^ (added to the revised manuscript).

2. and for the effectiveness of TV programs (Such as cartoons or drama) in Bangladesh as well as other nations is described in Introduction section (line 73-82), and discussion (line 329 – 333) as well. That was one of the motivations for this manuscript, our theory is: if tv programs can be impactful on TB, HIV, smoking in Bangladesh, it also can be utilized for mental health promotion.

3. Empirical evidence consistently indicates that equitable distribution of household and cognitive tasks, alongside supportive policies to recognize and enable women’s autonomy, leads to improved decision-making power, psychological well-being, and health-seeking behaviors among housewives^5–8^ (added to the revised manuscript)

**#7a.** Consider prioritizing recommendations in order of impact and feasibility

**#7b.** Frame future research as part of a broader national mental health strategy

Our Response: Both sections are revised and re-structured accordingly.

**#8** Capitalization has been used unnecessarily within sentences, for example, in phrases like “Strata,” “Smartphone,” “Internet,” “Newspaper/Magazine,” “Radio,” “Television,” “Age,” “Education Level,” “Occupation,” “Marital Status,” “Religion,” and “Media Exposure”, which disrupts consistency and proper grammar.

Our Response: We have proof-read the whole manuscript again, and corrected these typos and inconsistencies from us in the revised manuscript.

## **Comments from Reviewer 2:**

**#1** This is a well-designed study that promotes gender equity and inclusion in design of mental health programmes in resource poor settings. It was observed that 92.4% of respondents were in a particular religious inclination and therefore it may be difficult to analyse the influence of religion on the care seeking behaviour of these women. In subsequent studies, the authors may wish to consider exploring the role of religion on their study outcomes or possibly compare their findings with those of women from other socio-cultural (religious) settings. This is an important factor that the authors may wish to consider.

Our Response: Well, Bangladesh is predominantly a Muslim country. The 2022 national census reported that nearly 91% of Bangladesh’s population are Muslims. Given this religious homogeneity that matches with the proportion of our findings, we did not consider religion as a primary explanatory factor in our analysis, nor did we hypothesize any specific theoretical pathways linking religion to mental health help-seeking behavior.

# **Citations:**

1.	Kaaya S, Siril H, Fawzi MCS, et al. A peer-facilitated psychological group intervention for perinatal women living with HIV and depression in Tanzania-Healthy Options: A cluster-randomized controlled trial. *PLoS Med*. 2022;19(12):e1004112. doi:10.1371/journal.pmed.1004112

2.	Ara G, Khanam M, Papri N, et al. Peer counselling improves breastfeeding practices: A cluster randomized controlled trial in urban Bangladesh. *Matern Child Nutr*. 2018;14(3):e12605. doi:10.1111/mcn.12605

3.	Topping KJ. Peer Education and Peer Counselling for Health and Well-Being: A Review of Reviews. *Int J Environ Res Public Health*. 2022;19(10):6064. doi:10.3390/ijerph19106064

4.	Bolton P, Bass J, Neugebauer R, et al. Group Interpersonal Psychotherapy for Depression in Rural UgandaA Randomized Controlled Trial. *JAMA*. 2003;289(23):3117-3124. doi:10.1001/jama.289.23.3117

5. Olwanda E, Opondo K, Oluoch D, et al. Women’s autonomy and maternal health decision making in Kenya: implications for service delivery reform - a qualitative study. *BMC Womens Health*. 2024;24(1):181. doi:10.1186/s12905-024-02965-9

6. Zegeye B, Idriss-Wheeler D, Ahinkorah BO, et al. Association between women’s household decision-making autonomy and health insurance enrollment in sub-saharan Africa. *BMC Public Health*. 2023;23(1):610. doi:10.1186/s12889-023-15434-z

7. The Impact of Household Structure on Female Autonomy in Developing Countries: The Journal of Development Studies: Vol 51 , No 5 - Get Access. Accessed August 5, 2025. https://www.tandfonline.com/doi/full/10.1080/00220388.2014.983909?utm_source=chatgpt.com

8. Kumar N, Quisumbing A, Manohar S, et al. Intrahousehold dynamics in South Asia:

understanding the relationships among women’s empowerment, task sharing, women’s

decision making, and diets. Published online January 9, 2025:2025.01.08.25320196.

doi:10.1101/2025.01.08.25320196
